# An experimental investigation of spin-on doping optimization for enhanced electrical characteristics in silicon homojunction solar cells: Proof of concept

**DOI:** 10.1016/j.heliyon.2024.e31193

**Published:** 2024-05-14

**Authors:** Ili Salwani Mohamad, Pin Jern Ker, Puvaneswaran Chelvanathan, Mohd Natashah Norizan, Boon Kar Yap, Sieh Kiong Tiong, Nowshad Amin

**Affiliations:** a Department of Electrical and Electronic Engineering, Universiti Tenaga Nasional, Kajang, 43000, Malaysia; b Universiti Malaysia Perlis (UniMAP), Arau, 02600, Malaysia; c Institute of Sustainable Energy, Universiti Tenaga Nasional (UNITEN), Kajang, 43000, Selangor, Malaysia; d Solar Energy Research Institute, Universiti Kebangsaan Malaysia, Bangi, 43600, Malaysia; e Geopolymer and Green Technology, Centre of Excellent (CEGeoGTech), Universiti Malaysia Perlis (UniMAP), Arau, 02600, Malaysia; f Dept. of Electrical and Electronic Engineering, American International University-Bangladesh (AIUB), Kuril, Dhaka, 1229, Bangladesh; g School of Engineering and Technology, Sunway University, Bandar Sunway 47500, Malaysia

**Keywords:** Energy, Solar cell, Silicon, Homojunction, Emitter thickness, Spin on doping, Series resistance, Shunt resistance, Efficiency

## Abstract

The pursuit of enhancing the performance of silicon-based solar cells is pivotal for the progression of solar photovoltaics as the most potential renewable energy technologies. Despite the existence of sophisticated methods like diffusion and ion implantation for doping phosphorus into p-type silicon wafers in the semiconductor industry, there is a compelling need to research spin-on doping techniques, especially in the context of tandem devices, where fabricating the bottom cell demands meticulous control over conditions. The primary challenge with existing silicon cell fabrication methods lies in their complexity, cost, and environmental concerns. Thus, this research focuses on the optimization of parameters, such as, deposition of the spin on doping layer, emitter thickness (X_j_), and dopant concentration (N_D_) to maximize solar cell efficiency. We utilized both fabrication and simulation techniques to delve into these factors. Employing silicon wafer thickness of 625 μm, the study explored the effects of altering the count of dopant layers through the spin-on dopant (SOD) technique in the device fabrication. Interestingly, the increase of the dopant layers from 1 to 4 enhances efficiency, whereby, further addition of 6 and 8 layers worsens both series and shunt resistances, affecting the solar cell performance. The peak efficiency of 11.75 % achieved in fabrication of 4 layers dopant. By using device simulation with wxAMPS to perform a combinatorial analysis of X_j_ and N_D_, we further identified the optimal conditions for an emitter to achieve peak performance. Altering X_j_ between 0.05 μm and 10 μm and adjusting N_D_ from 1e+15 cm^−3^ to 9e+15 cm^−3^, we found that maximum efficiency of 14.18 % was attained for X_j_ = 1 μm and N_D_ = 9e+15 cm^−3^. This research addresses a crucial knowledge gap, providing insights for creating more efficient, cost-effective, and flexible silicon solar cells, thereby enhancing their viability as a sustainable energy source.

## Highlights

• Optimized efficiency in rudimentary silicon solar cell structure using spin-on doping methodology with different dopant amounts. The increment of the dopant amount improves efficiency up to a point beyond which the performance degrades.
• Reduced temperature of drive-in diffusion with dynamic nitrogen flow.
• Fine-tuning on thickness and carrier concentration for silicon bottom cell for potential tandem configuration.
• Comprehensive empirical and simulation analysis.

## Graphical abstract

**Figure:**
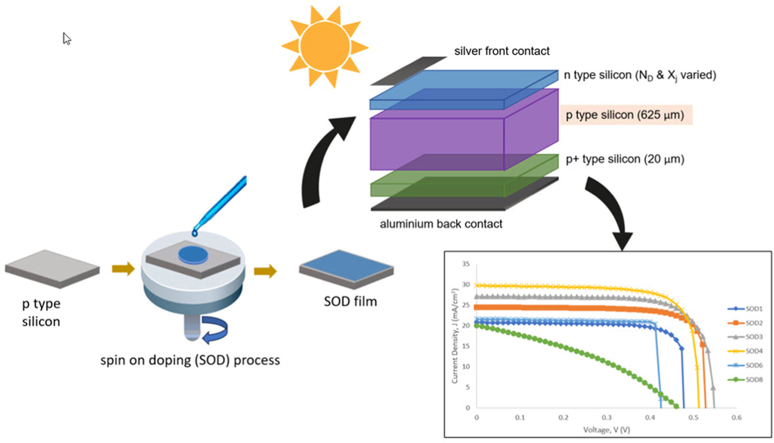

## 1 Introduction

As a beacon of sustainable energy solutions, solar power is becoming increasingly important in today's world of rising energy demands and mounting environmental concerns. The urgency of reducing anthropogenic carbon emissions emphasizes the importance of efficiently harnessing solar energy. Solar cells, which use the photovoltaic effect to generate electricity, promise a clean and sustainable source of energy. Silicon solar cells dominate the photovoltaic market, currently accounting for more than 95 % of the total, overshadowing thin film technologies that include CdTe and CIGS [1]. Their dominance is due to the abundance of silicon (second only to oxygen in the earth's crust), its non-toxicity [2], environmental friendliness, and remarkable stability, which exhibits minimal degradation over time [3]. The current silicon homojunction device is utilizing passive emitter and rear contact cells (PERC) on a p-type crystalline silicon wafer. According to the National Renewable Energy Laboratory (NREL), the peak performance of single homojunction p-type PERC cells, with a notable exception from the University of New South Wales (UNSW), has been recorded at 25 % under the global AM 1.5 spectrum (1000 W/m^2^) [4]. However, with advancements such as texturization, anti-reflective coatings, and passivation layers, this can potentially reach the theorized limit of ∼30 % predicted by Shockley-Queisser for single-junction solar cells [5].

Given the pivotal role of silicon solar cells in the photovoltaic market, their application as the bottom cell in tandem configurations further underscores their significance. Tandem solar cells, which stack multiple photovoltaic layers to capture a broader spectrum of sunlight, can surpass the efficiency limits of single-junction cells. In these configurations, the silicon bottom cell is crucial for absorbing lower-energy photons, while the top cell captures higher-energy photons. This synergistic approach aims to elevate overall efficiency beyond the single-junction limit. The meticulous attention to silicon fabrication steps is paramount in tandem cell construction due to the delicate balance required to optimize the overall device performance. Any imperfections in the silicon bottom cell, such as defects or impurities, can significantly impact the tandem cell's efficiency. Moreover, the optical and electrical properties of the silicon layer must be finely tuned to complement the top cell, ensuring maximum light absorption and minimal energy loss. This necessitates advanced fabrication techniques and rigorous quality control to maintain the integrity of the silicon substrate, making the fabrication process both a critical and challenging endeavor in the development of high-efficiency tandem solar cells.

A planar diode structure is fundamental to the architecture of crystalline silicon-based solar cells. This configuration consists of a thin, heavily doped silicon layer (either n + or p+) on the front surface of a moderately doped silicon wafer of the opposite type (either p or n) [6]. This heavily doped layer, known as the emitter, forms a pivotal p-n junction interface, facilitating the separation of photo-generated electron-hole pairs and converting absorbed sunlight into electrical energy. This layer's quality and efficacy are critical to the overall performance of the solar cell. While traditional doping techniques, such as ion implantation or gas-phase diffusion, have been the mainstay for forming emitters, they have their own set of challenges. These methods frequently necessitate high-temperature processes, utilize intricate equipment, and face scalability and reproducibility challenges. Spin-on doping (SOD) is a promising, cost-effective alternative that possesses simplicity as executed in a non-vacuum environment to be environmentally friendly and adaptable across various substrates and architectures.

The SOD method uses a non-hygroscopic solution that is spun onto the surface of the semiconductor substrate and then baked to create a uniform distribution of dopants across the substrate. The process provides precise control of dopant concentrations in the silicon substrate and combines two furnace processes into one. Martinez et al. reported that a homojunction silicon solar cell with a textured silicon surface produced a stable 14 % efficiency on an active area of 100 mm^2^ using a traditional diffusion furnace under ambient conditions [7]. Similarly, Gangopadhyay et al. demonstrated efficiencies within a comparable range using the SOD method for emitter formation on their textured silicon solar cell, which was also equipped with an anti-reflecting coating [8]. Moreover, the commercial viability of homojunction silicon cells, especially in tandem designs, has been further validated by a recent study by Kim et al. In this study, spin-on doping was utilized for emitter formation in the bottom silicon cell, which was paired with a perovskite layer to enhance spectral absorption coverage. The standalone silicon cell performance achieved an efficiency of 12.8 %, while the two-terminal tandem design yielded an impressive 21.19 % efficiency [9].

Despite these benefits, the comprehensive optimization of SOD parameters, particularly for phosphorus-doped emitters in silicon-based homojunction solar cells, has received little attention. Dopant homogeneity on wafer surfaces poses a challenge, influencing junction formation. Non-uniformities in electrical characterization can be caused by factors such as dopant viscosity, spin rotation, and spin speed [10]. Ding et al. demonstrated the effectiveness of phosphorus doping in polycrystalline silicon using the SOD technique, resulting in improved passivating contacts for silicon solar cells. Nonetheless, the research encountered significant challenges, particularly in attaining a uniform dopant distribution and managing thermal processing for dopant activation [11]. In the other study, Yang et al. confronted significant challenges, particularly in achieving precise control over dopant penetration depth and ensuring complete activation of the dopants while exploring the augmentation of solar cell efficiency through the integration of SiO_2_/poly-Si layers using SOD [12]. In the context of enhancing solar cell efficiency, the Fraunhofer Institute for Solar Energy Systems (ISE) has conducted pivotal research exploring various doping techniques, notably including spin-on doping. A critical aspect of this investigation focuses on the potential integration of SOD into large-scale production, a process currently impeded by the challenge of maintaining consistent doping levels across different production batches.

Recognizing the identified knowledge gap, the study conducts an in-depth investigation of spin-on doping parameters for phosphorus dopants in the emitter formation of homojunction-based silicon solar cells. This research looks into the practical fabrication techniques of the silicon solar cells, varying SOD layers with optimized spinning speed and diffusion parameters. In addition, computational simulations are used to manipulate the key parameters such as dopant concentration (N_D_) and emitter junction depth/thickness (X_j_) in order to understand their impact on key solar cell performance indicators such as open-circuit voltage, short-circuit current, and eventually the conversion efficiency.

## 2 Experimental procedure

### 2.1 Fabrication method

#### 2.1.1 Silicon solar cells with varied SOD layers

**Fig. 1:**
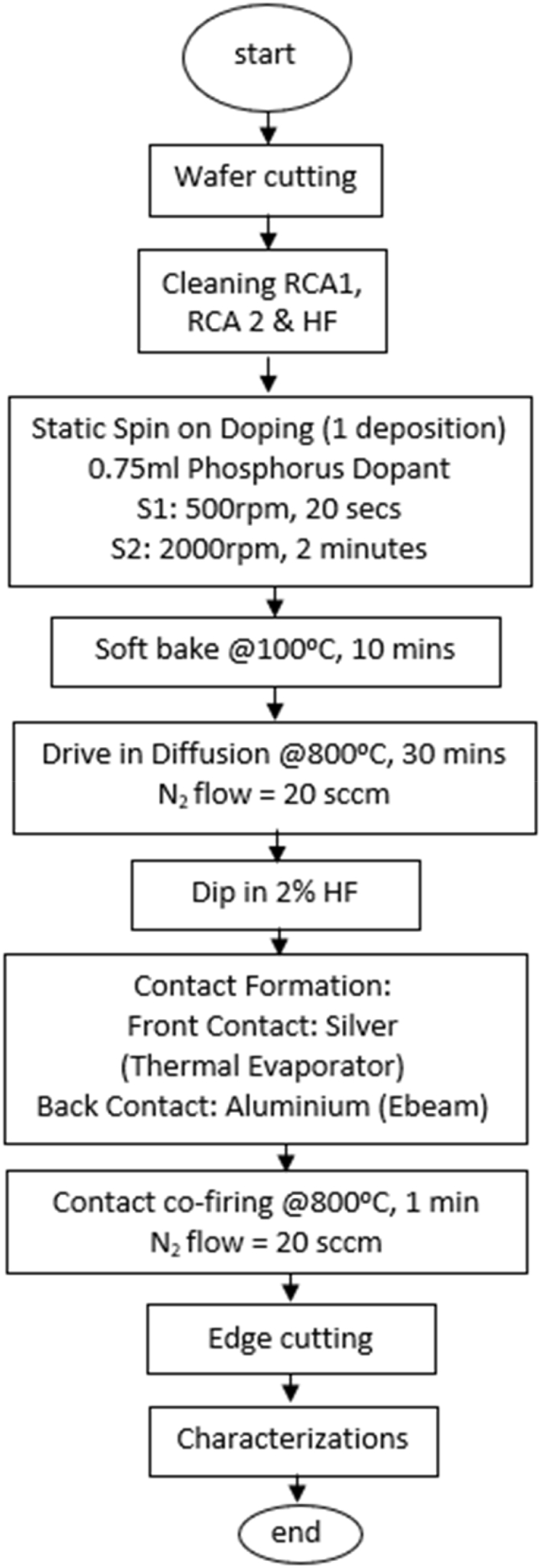
Overall flowchart for silicon solar cell fabrication followed in the study.

In this study, the homojunction silicon solar cells were fabricated on a double-sided polished p-type (100) monocrystalline Si wafer sourced from Czochralski (CZ) processed ingots. The resistivity of the Si wafer was 0.04 Ω cm, and the thickness was 625±10 μm. Fig. 1 shows the overall fabrication flowchart of this work. The Si substrate was cut to 4 cm × 4 cm dimensions before being cleaned using standard RCA cleaning procedures. The sequential application of RCA1, RCA2, and a 2 % hydrofluoric (HF) acid dip removed organic residues, metal ions, and native silicon oxide from the silicon surface, respectively.

**Fig. 2:**
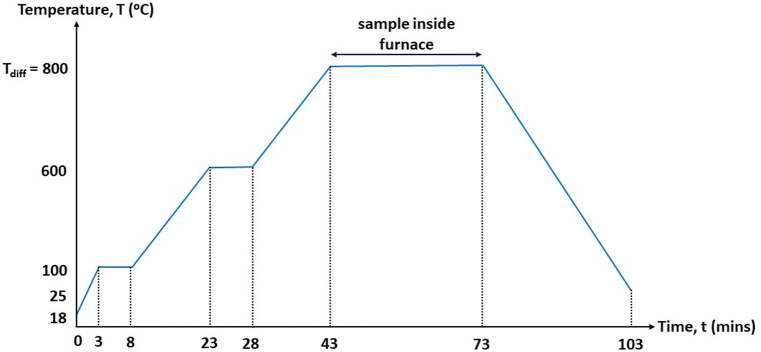
Drive-in diffusion temperature profile.

Subsequently, an amount of 0.75 ml phosphorus (PDC5-2500, sourced from Futurrex using an IKA micropipette, was applied onto the silicon substrate, sample denoted as SOD1, using a Laurell (model WS-650Hz-23NPPB) spin coater. The SOD parameters used involved a spinning rate of 500 rpm for 20 s, followed by 2000 rpm for 2 min. The phosphorus-coated sample was then soft-baked for 10 min on an IKA C-MAG HS7 digital hot plate. The SOD1 sample was then driven-in diffused at 800 °C for 30 min in a nitrogen-rich (20 sccm dynamic flow), closed tube environment using the thermal annealing system (Kenix Corporation, Japan; model KAN80). This phase targeted the incorporation of phosphorus dopant into the silicon, substituting phosphorus for inner substrate atoms and resulting in an n-type layer. Importantly, electrical phosphorus activation occurs primarily between 700 °C and 900 °C, which corresponds to activation temperatures during post-implantation anneal of non-amorphized silicon layers, as described in Ref. [13]. The drive-in diffusion temperature profile is essential to control the depth and distribution of dopants within the silicon wafer, influencing the electrical characteristics of the solar cell. Fig. 2 illustrates the drive-in temperature profile for this work. The temperature is increased in stages before being maintained at the desired level to allow for a gradual and controlled diffusion process. This staged increment helps to optimize the dopant diffusion profile, ensuring uniformity and precision in the doping process, which is crucial for achieving the desired electrical performance and efficiency of the solar cell. The duration of each temperature stage is determined by the equipment's ramp rate, which is 33 °C/min.

The subsequent annealing procedure precipitated the formation of phosphosilicate glass (P_2_O_5_) on the silicon surface, which was carefully removed by immersing the samples in a 2 % HF solution until the silicon surface exhibited its characteristics. The phosphosilicate glass layer must be effectively removed, as this insulating layer has the possibility to restrict device performance if not adequately cleaned. Metallization processes were then done using a thermal evaporator and e-beam evaporator system (sourced from Leader Advanced Technology company). The front and rear contacts for the silicon solar cells incorporated both silver (Ag) and aluminum (Al) based on their preferred work functions. The front contact was designed with finger-like electrodes covering less than 10 % of the total cell area to allow for sufficient light absorption through the front surface of the cell, facilitating comprehensive carrier collection across the entire sample area [14]. Following the electrode formation, the sample was co-fired in the thermal annealing system at 800 °C for 1 min in a closed environment with a continuous 20 sccm nitrogen gas flow and pressure of 0.5 torr. After cooling, the samples were resized into 2 cm × 2 cm dimensions to eliminate edge-related discrepancies, such as potential electrical shunting caused by uneven doping concentrations near the edges [15].

**Fig. 3:**
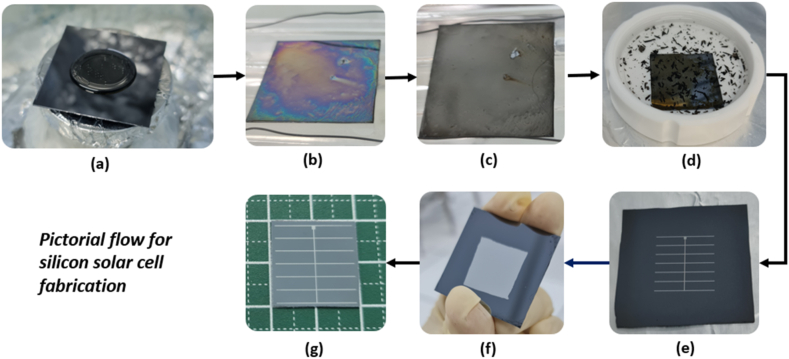
(a) Sample with SOD phosphorus deposition, (b) sample after soft-bake, (c) sample after drive-in diffusion, (d) sample after phosphosilicate layer removal, (e) sample with silver electrode deposition, (f) sample with aluminum deposition and (g) silicon solar cell after edge isolation process. (For interpretation of the references to colour in this figure legend, the reader is referred to the Web version of this article.)

The fabrication procedure was replicated for samples SOD2, SOD3, SOD4, SOD6, and SOD8, denoting the application of 2, 3, 4, 6, and 8 phosphorus dopant layers, respectively, spun onto a p-type silicon substrate. It is important to note that these layer variations were only implemented during the SOD phase, whereby subsequent SOD layers were added after the soft-bake phase. This methodology was used consistently until the desired layer counts were achieved. Fig. 3 concisely outlines the key stages in the fabrication of silicon solar cells, from dopant deposition to final cell formation. It begins with phosphorus deposition on the silicon wafer (a), followed by a soft-bake to prepare for drive-in diffusion (b), where dopants are thermally incorporated into the substrate to form the n-type layer (c). The subsequent removal of the phosphosilicate glass layer (d) is crucial for maintaining the cell's performance. Metallization is depicted with silver electrode deposition on the front (e) and aluminum on the back (f), optimizing light absorption and electrical contact. The process concludes with edge isolation (g) to enhance uniformity and eliminate potential shunting, underscoring the precision required in each step to achieve efficient solar cell performance.

#### 2.1.2 Characterization methods

The fabricated silicon solar cells with distinct spin-on doping layers and dopants underwent various necessary characterizations. Raman spectroscopy was used to investigate the presence of phosphosilicate glass in pre–HF–dipped samples. Moreover, Hall effect measurement was conducted to look at the comprehensive evaluation of the silicon wafers' electronic properties to correlate with the fabricated solar cell characteristics. Electrical parameters, such as short circuit current density (J_sc_), open circuit voltage (V_oc_), fill factor (FF), and efficiency (ɳ), were carefully measured using the Oriel Sol3A Solar Simulator. This characterization was executed under AM 1.5G solar spectrum illumination, targeting 1000 W/m^2^ at the front surface. A balance, or trade-off, between these parameters is crucial. The equation is as follows;

(1) $$ɳ=\frac{P_{out}}{P_{in}}=\frac{J_{sc}xV_{oc}xFF}{P_{in}}$$

synthesizes this interdependence. Here, η represents the efficiency, P_out_ is the output power, P_in_ denotes the incident power, J_sc_ is the short-circuit current density, V_oc_ signifies the open-circuit voltage, and FF stands for the fill factor.

### 2.2 Simulation studies

#### 2.2.1 Device Modelling

Silicon wafer thickness, doping concentration, defect density, and working temperature are all physical characteristics that have a significant impact on solar cell performance [16]. The outcome of numerical simulation provides a powerful mechanism for uncovering the intricate relationship between constructing layer attributes and solar cell behavior. This method enables an economical and efficient strategy for probing design alternatives, refining parameters, gaining insights into device behavior, and streamlining decision-making processes. By integrating with experimental initiatives, numerical simulation significantly reduces the need for exhaustive trial and error, resulting in tangible benefits in time and resource management.

**Fig. 4:**
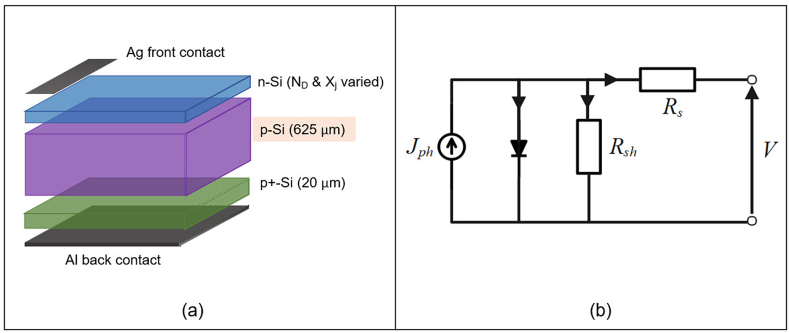
(a) Silicon solar cell schematic structure and (b) Equivalent circuit for silicon solar cell. (For interpretation of the references to colour in this figure legend, the reader is referred to the Web version of this article.)

The wxAMPS-1D (AMPS: Analysis of Microelectronic and Photonic Structures) numerical simulation software was used in this study to assess the effect of emitter thickness and phosphorus doping concentration on the electrical performance of the silicon solar cell. The software's graphical user interface (GUI), created with the cross-platform C++ library wxWidgets, allows for quick data input while also enhancing visualization capabilities for result comparison and analysis. It includes tunneling models, intra-band tunneling, and trap-assisted tunneling and takes into account the effects of series and shunt resistances outside of the primary diode. Notably, the wxAMPS algorithm has been improved to combine the Newton and Gummel methodologies, improving convergence and stability. Besides, this software incorporates three fundamental equations to represent the physics of device transport: Poisson's equation, the continuity equation for free holes, and the continuity equation for free electrons. Fig. 4 depicts the proposed baseline silicon solar cell structure, as well as its equivalent circuit. Simulated devices were illuminated under the AM 1.5G solar spectrum through the frontal layers of solar cells.

Table 1 summarizes the cell parameters used, which were derived from theoretical frameworks, experimental data, and existing literature. The focal variations included N_D_ (emitter carrier concentration) and X_j_ (junction depth or emitter thickness). N_D_ = 1e+15 to 9e+15 cm^−3^ (incremented by 1e+15) and X_j_ = 0.05 μm, 0.1 μm–10 μm (incremented by 1 μm) were the prescribed variation ranges. For this study, it is important to mention that the inputs for defect (band tails), Go (conduction, valence), SigN (conduction, valence), and SigP (conduction, valence) were marked as "na" (not applicable) in the software used.

**Table 1:** Physical and electronic properties of materials/layers used in the simulation.

Material properties	n-Si	p-Si	p^+^-Si
Thickness (μm)	varied	625	20.000
Dielectric permittivity ɛ/ɛ_0_	11.9	11.9	11.9
Bandgap (E_g_) (eV)	1.124	1.124	1.124
Electron affinity χ (eV)	4.05	4.05	4.05
C_B_ density of state (x10^19^) (cm^−3^)	2.85	2.85	2.85
V_B_ density of state (x10^19^) (cm^−3^)	1.04	1.04	1.04
Electron mobility (cm^2^ V^−1^s^−1^)	1350	1350	1350
Hole mobility (cm^2^ V^−1^s^−1^)	450	450	450
N_D_ density (cm^−3^)	varied	0	0
N_A_ density (cm^−3^)	0	1e+16	1e+18

For each layer thickness, configurations such as mesh grid "Edge" and "Center" were meticulously adjusted under the "Advanced" tab, adhering to developer guidelines and the methodology exemplified in Ref. [17]. Equations (2), (3)) provide the necessary calculations in cases where the variable *x* must be satisfied:

(2) $$Grid\;Edge\;\to \;\frac{Total\;thickness\;\left(nm\right)\;}{x}=5000\;or<8000$$

(3) $$Grid\;Centre\;\to \;\frac{Total\;thickness\;\left(nm\right)\;}{x}=250$$

In order to analyze and predict electrostatic carrier transport behaviors in various systems, Poisson's and continuity equation can be utilized. Poisson's equation, as in (4), connects spatial charge distribution with the electric potential, providing insights into the electrostatic landscape, including the electric potential and derived electric fields.

(4) $$\frac{\partial }{\partial x^{2}}\emptyset \left(x\right)=\frac{q}{\varepsilon }\left[n\left(x\right)-p\left(x\right)\right]$$

where ∅ is the electric potential, q is the elementary charge, ε is the dielectric constant, n, and p refer to electron and hole density, respectively. The continuity equation, as in (5), describes the behavior and flow of charge carriers, encompassing processes like generation, recombination, diffusion, and drift, thus offering insights into current flow and the response of carriers to various stimuli. It describes the current intensity distribution in space.

(5) $$+\frac{1}{q}\;\frac{\partial }{\partial x}J_{n}\left(x\right)=G\left(x\right)-R\left(x\right)$$

$$-\frac{1}{q}\;\frac{\partial }{\partial x}J_{p}\left(x\right)=G\left(x\right)-R\left(x\right)$$

where R is the recombination rate, and G is the generation rate. $J_{n,p}$ represents electron and hole current density, respectively. Coupling the Poisson and continuity formulas, equation (6) can be deduced, which guarantees that the total current density is a constant anywhere in the area.

(6) $$\frac{\partial }{\partial x}\left[J_{n}\left(x\right)+J_{p}\left(x\right)\right]=0$$

In addition to that, the drift-diffusion equation as in (7) can be used to connect Poisson's equation and current continuity equations, which is worth noting that, in the equilibrium state, the total current density, J is position-independent.

$$J_{n}\left(x\right)=-qn\left(x\right)\mu_{n}\left(x\right)\frac{\partial }{\partial x}\emptyset \left(x\right)+qD_{n}\left(x\right)\frac{\partial }{\partial x}n\left(x\right)$$

$$J_{p}\left(x\right)=-qp\left(x\right)\mu_{p}\left(x\right)\frac{\partial }{\partial x}\emptyset \left(x\right)+qD_{p}\left(x\right)\frac{\partial }{\partial x}p\left(x\right)$$

(7) $$J\left(x\right)=J_{n}\left(x\right)+J_{p}\left(x\right)$$

where $\mu_{n,p}$ is the carrier mobility and $D_{n,p}$ is the carrier diffusion coefficient of electrons and holes, respectively.

## 3 Results and discussion

### 3.1 Experimental observation

#### 3.1.1 Raman spectroscopy

**Fig. 5:**
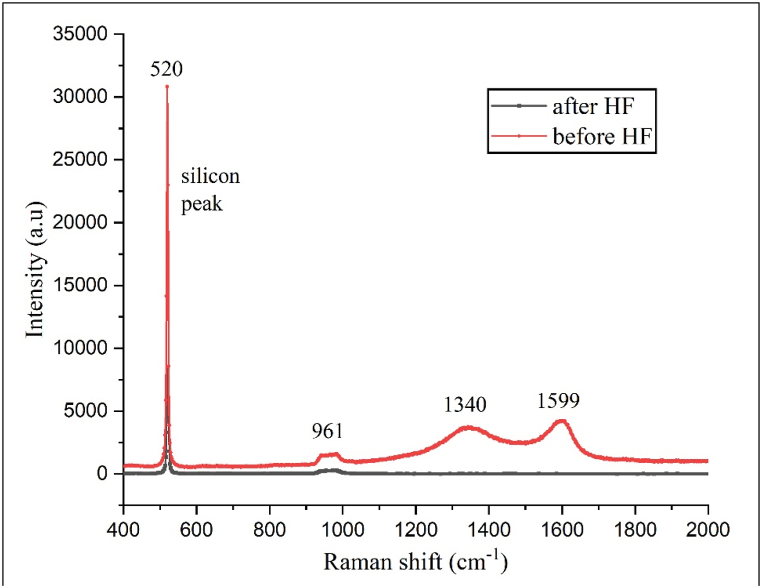
Raman spectra of phosphosilicate glass layer before and after HF cleaning.

During the fabrication phase of this study, homojunction silicon solar cells with variations in the phosphorus dopant layer deposition used in the spin-on doping process were developed. Different cells were evaluated, each with a different number of layers, such as 1, 2, 3, 4, 6, and 8. Samples are identified as SOD1, SOD2, SOD3, SOD4, SOD6, and SOD8, corresponding to their counts of spin-on doping layers. Raman spectroscopy is used to study the vibrational modes of molecules and materials. As illustrated in Fig. 5, regarding the formation of phosphosilicate glass following the drive-in diffusion process, the peak at 961 cm⁻^1^ corresponds to the symmetric stretching vibration of the Si–*O*–Si bond within the silicate network [18]. The 1340 cm⁻^1^ peak is tied to the symmetric stretching vibration of the P–O bond in the phosphate group [18]. In contrast, the 1599 cm⁻^1^ peak is linked to the asymmetric stretching vibration of the Si–*O*–Si bond in the silicate structure [18]. The 520 cm⁻^1^ peak relates to the silicon substrate; following an HF dip, the phosphosilicate glass is removed, leaving only the silicon peak evident. The observed Raman peaks provide insights into the structure and composition of the phosphosilicate glass. The intensity of the Raman peak at 940 cm⁻^1^ escalates with increasing ytterbia content in the glass. Similarly, the Raman peak at 961 cm⁻^1^ becomes more pronounced with higher SiO_2_ content in the glass but diminishes after a certain threshold, as also verified in Ref. [19].

#### 3.1.2 Current Density–Voltage characterization

**Fig. 6:**
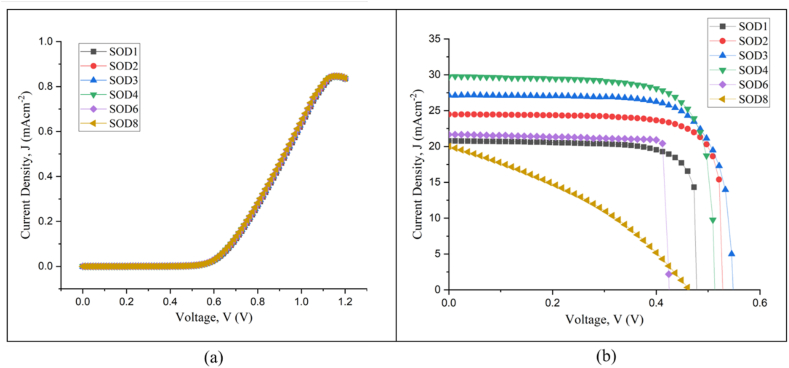
(a) Dark J-V and (b) Light J-V curves for all SOD layer variations tested.

**Fig. 7:**
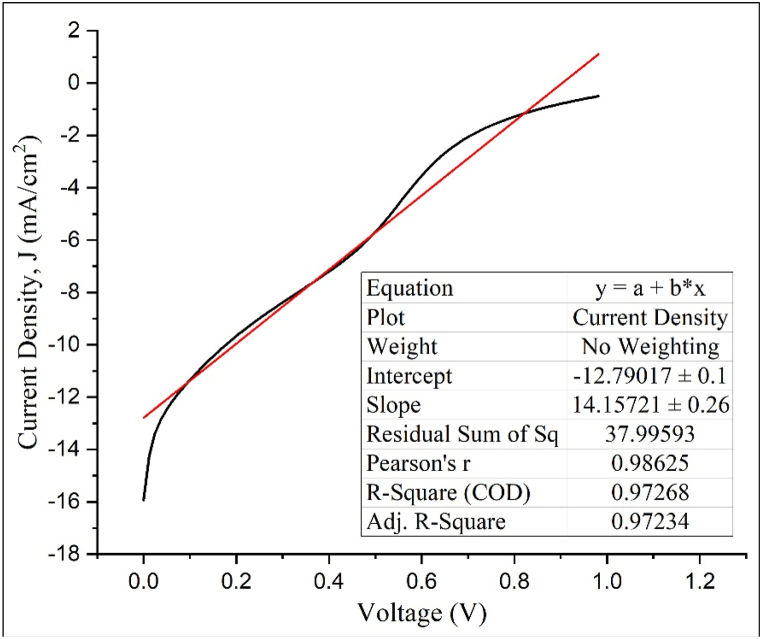
Dark J-V characteristics fitted to the single-diode model.

Fig. 6 demonstrates the current density-voltage (J-V) characteristics fundamental to the performance metrics of solar cells, including homojunction silicon solar cells. The dark J-V curves in Fig. 6(a) expose the inherent diode behavior of these cells in the dark, shedding light on critical processes like recombination and carrier transport. The ideality factor (n) quantifies how closely the behavior of an actual solar cell or diode mirrors that of an ideal one, with a particular emphasis on the recombination processes within the depletion zone. Ideally, the generation and recombination of charge carriers, such as electrons and holes, should proceed solely through diffusion. Expanding on this, Fig. 7 presents a linear fit of ln(J) versus V from the dark J-V data, focusing on a specific linear region for analysis. The linear portion of the J-V curve in the forward bias region (positive voltage values) where the current density (J) increases exponentially with voltage (V). This is typically done before the current starts to saturate due to series resistance effects. The slope of this line is related to the ideality factor through the thermal voltage ($V_{T})$, where $V_{T}=\frac{kT}{q}$, k is the Boltzmann's constant, T is the absolute temperature in Kelvin, and q is the charge of an electron.

(8) $$n=\frac{1}{S.V_{T}}$$

where;

S = slope of the linear fit.

$V_{T}$ = thermal voltage

Given that $V_{T}=\frac{kT}{q}$; and substituting the constants ($k=1.381\;x\;10^{-23}J/K$, $q=1.602\;x\;10^{-19}C$), for room temperature (T≈298K), $V_{T}\approx 25.95\;mV)$.

The ideality factor of 2.73 was obtained using the slope, S, which is 14.16 V^-1^ in this case from the linear fit and the thermal voltage, V_T_. Values greater than 2 can indicate more complex recombination mechanisms, such as tunneling or surface recombination, or may reflect non-idealities like series or shunt resistances, leading to potential losses in solar cell efficiency.

Notably, all tested samples exhibit an ideal p-n junction diode curve with a built-in potential (V_bi_) of approximately 0.65 V. The light J-V curves, shown in Fig. 6 (b), illuminate the cells' performance under operational or illuminated conditions. The metrics derived from these curves, summarized in Table 2, shed light on the efficiency of each sample in converting solar energy into electricity. According to the data, the J_sc_ has a direct relationship with the capacity of the solar cell to convert absorbed photons into electron-hole pairs. The highest J_sc_ of 29.76 mA/cm^2^ was observed in the SOD4 sample, which indicates superior light absorption and carrier generation.

**Table 2:** Detailed electrical properties of the tested silicon solar cells under illumination.

Sample	V_oc_, V	J_sc_, mA/cm^2^	FF, %	ɳ, %
SOD1	0.48	20.78	81.14	8.06
SOD2	0.53	24.49	80.32	10.39
SOD3	0.55	27.15	75.20	11.20
SOD4	0.51	29.79	76.92	11.75
SOD6	0.42	23.82	85.68	8.67
SOD8	0.46	20.03	36.01	3.34

In contrast, the SOD8 sample, with the lowest J_sc_ of 20.02 mA/cm^2^, suggests a deteriorated ability of light absorption or carrier transport or both. The V_oc_ represents the potential difference between the quasi-Fermi levels of electrons and holes [20]. It is proportional to the rate of recombination within the cell. The peak V_oc_ of 0.55 V in the SOD3 sample indicates minimal recombination, whereas the lower V_oc_ of 0.42 V in the SOD6 sample could indicate enhanced recombination events. The FF, which reflects the "squareness" of the J-V curve, is an indicator of the cell's operational efficiency. The SOD6 sample has the highest FF of 85.68 %, indicating efficient charge separation and minimal recombination. When all of the metrics mentioned earlier are combined, the SOD4 sample has an overall efficiency of 11.75 %. Surprisingly, increasing the number of dopant layers from 1 to 4 improves efficiency, whereas the trend reverses beyond the limit, such as for 6 and 8 layers. This decrease is supported by an increase in both shunt and series resistances, as shown in Fig. 6 (b), which could be due to complications caused by excessive doping or structural defects. Hence, while increasing dopant layers in the SOD process initially improves solar cell performance, there is an optimal limit beyond which performance degrades.

#### 3.1.3 Hall effect measurement of silicon samples

Hall measurements are an essential tool for determining the electrical properties of semiconductor materials. The data presented in Table 3 reveal intriguing behaviors across the various dopant layer variations. The persistent negative sign-in sheet concentration values are consistent with observation across all samples. This demonstrates the successful conversion of silicon from its natural p-type base to an n-type configuration. The relative uniformity of these values across dopant layers suggests a saturation phenomenon. This means that even with just one dopant layer, the silicon may have reached its doping limit. As a result, additional layers may not significantly increase carrier concentration beyond this saturation threshold. When compared to efficiency trends, it is clear that the increase in efficiency from 1 to 4 layers can be attributed to reaching an optimal doping threshold. This most likely reduces recombination events, thereby increasing the electric field at the junction. However, the decrease in efficiency observed for the 6 and 8-layer configurations suggests that doping beyond the optimal threshold is counterproductive. This can introduce defects, boost recombination centers, or cause "carrier freeze-out," in which dopants congregate into inactive clusters [21]. Excessive doping can increase series resistance or decrease shunt resistance, lowering fill factor and, as a result, reducing overall efficiency [22].

**Table 3:** Electrical properties obtained from Hall effect measurements for the tested samples.

SOD	Bulk Concentration (cm^−3^)	Sheet Concentration (cm^−3^)	Sheet Resistance (Ω)	Resistivity (Ωm)	Conductivity (S)	Mobility (m^2^V–^1^S^−1^)
Bare	2.61E+16	1.6329E+15	26.8192	1.6762	0.596586	142.542
SOD1	−5.6E+15	−3.494E+14	312.368	19.5232	0.051221	57.1968
SOD2	-5E+15	−3.098E+14	365.53	22.8456	0.043772	55.1188
SOD3	−2.7E+15	−1.689E+14	576.844	36.0528	0.027737	64.0784
SOD4	−5.1E+15	−3.211E+14	352.86	22.0536	0.045344	55.0938
SOD6	−3.8E+15	−2.39E+14	355.132	22.1958	0.045053	73.5646
SOD8	−4.7E+15	−2.954E+14	345.112	21.5696	0.046362	61.2234

The lack of a clear trend in conductivity and mobility as dopant layers vary could also be attributed to the physical limit, as mentioned earlier. Once silicon reaches doping saturation, subsequent dopant layers may not significantly alter these parameters. Furthermore, while Hall effect measurements are extremely useful, they may not capture all of the criteria of a material's electronic properties, especially if such variations are subtle [23,24]. As shown in Fig. 9 (b), efficiency improvements from 1 to 4 dopant layers may be a manifestation of an ideal doping level, optimizing recombination and carrier collection dynamics. The deteriorating trend observed at 6 and 8 layers suggests the complexities of semiconductor doping, where there is always an optimized limit for doping. This could be attributed to factors such as increased recombination, poor interface quality, or other phenomena not explicitly captured by Hall effect measurements [25]. Finally, the findings highlight the complex relationship between doping concentration and solar cell performance, revealing the fine balance required to optimize semiconductor devices.

### 3.2 Simulation outcomes

**Fig. 8:**
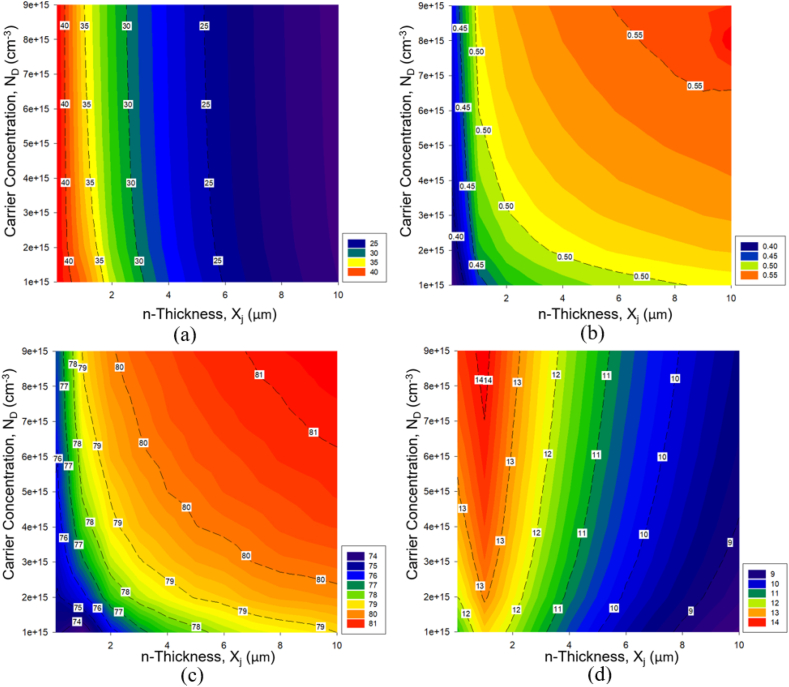
Simulation result of electrical parameters for homojunction silicon solar cell with respect to the variations of emitter carrier concentrations and thickness (a) J_sc_, (b) V_oc_, (c) FF, and (d) ɳ. (For interpretation of the references to colour in this figure legend, the reader is referred to the Web version of this article.)

Fig. 8 shows the simulation results for the electrical properties of the investigated homojunction silicon solar cell in terms of emitter carrier concentrations, N_D_, and emitter thickness (junction depth), X_j_. It finds an obvious decrease in short-circuit current density (J_sc_) with the increasing emitter thickness (X_j_). This trend can be attributed to the shorter distance carriers travel in a thinner emitter, which reduces the likelihood of recombination. A shallow junction depth reduces series resistance and increases J_sc_ by shortening the carrier path to the contact. This reaches a maximum of 41.79 mA/cm^3^ for the smallest X_j_ of 0.05 μm. A deeper junction, on the other hand, increases the carriers' traveling distance, increasing series resistance due to increased resistive losses. Notably, the J_sc_ value for a given X_j_ remains relatively consistent with the incremental N_D_. Fig. 8 (b) shows that the open-circuit voltage (V_oc_) is directly proportional to X_j_. Specifically, for all examined N_D_s, V_oc_ increases significantly from 0 μm to 1 μm, followed by a marginal increase. For N_D_ values ranging from 7e+15 cm^−3^ to 9e+15 cm^−3^, a peak in V_oc_ exceeding 0.55 V occurs at X_j_ = 10 μm. While a thinner emitter increases J_sc_ by providing a shorter path for carriers, it may increase surface recombination, potentially reducing V_oc_ [26].

A thicker emitter, on the other hand, can suppress surface recombination, increasing V_oc_. However, excessively thick emitter may aggravate bulk recombination, reducing carrier collection efficiency and eventually affecting J_sc_. The fill factor (FF), as shown in Fig. 8 (c), follows the V_oc_ trend, implying that as emitter thickness increases, the quality of the current-voltage characteristics curve improves. The observed pattern highlights efficient charge separation and reduced recombination in cells with thicker emitters. The highest value for FF in this context is 88.31 % at N_D_ = 9e+15 cm^−3^, with unity (100 %) representing the theoretical ideal. Finally, Fig. 8 (d) shows that the highest efficiency (ɳ) achieved in this study is 14.18 %, as the result of the synergetic optimization of J_sc_, V_oc_, and FF, as detailed in Table 4. The peak of the evaluated N_D_ values occurs at X_j_ = 1 μm, emphasizing the intricate balance between all performance metrics at this thickness. These findings, in essence, shed light on the intricate interdependence of solar cell design parameters and their collective role in shaping solar cell performance. When compared to previous research, these findings deepen our understanding of homojunction silicon solar cell behavior. Supplementary data provide an in-depth look at the electrical properties of the simulated cells in this study.

**Table 4:** Electrical performance of the optimized silicon homojunction solar cell.

Emitter Thickness, Xj (μm)	Carrier Concentration, ND (cm-3)	Current Density, Jsc (mA/cm-3)	Open Circuit Voltage Voc (V)	Fill Factor, FF (%)	Highest Efficiency, ɳ (%)
1	9.00e+15	35.05	0.512	79.06	14.18

#### 3.2.1 Quantum efficiency and current Density–Voltage characteristics of silicon solar cells

**Fig. 9:**
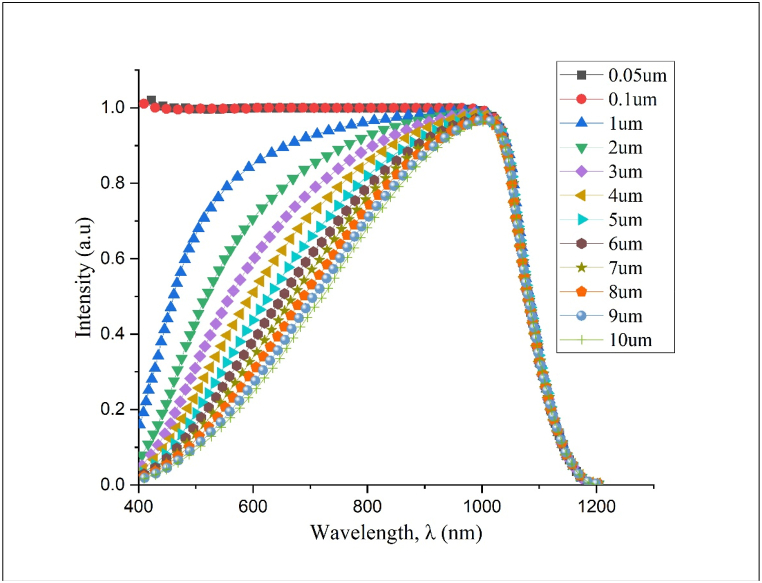
QE Spectra for silicon solar cells for all X_j_ variations at N_D_ = 9e+15 cm−^3.^(For interpretation of the references to colour in this figure legend, the reader is referred to the Web version of this article.).

Fig. 9 shows the quantum efficiency (QE) spectra for silicon solar cells with a doping concentration of N_D_ = 9e+15 cm^−3^ across various emitter thicknesses as a continuation of the investigation into the electrical properties of silicon solar cells. It is found that the junction depth (X_j_) or emitter thickness plays an important role in photon absorption and has a direct impact on device efficiency. Superior QE coverage is clearly observed for cells with emitter thicknesses of X_j_ = 0.05 μm & 0.1 μm. This strong quantum efficiency can be attributed to the shorter diffusion length of the generated minority carriers in thinner emitters, which facilitates more efficient photon absorption near the top surface. This increased photo-generated carriers result in increased quantum efficiency and, as a result, a higher J_sc_. Solar cells with an emitter thickness of X_j_ = 1 μm, on the other hand, represent the pinnacle of device optimization in this study. This efficiency optimization is based on a distinct trade-off between J_sc_ and V_oc_. While thinner emitters increase J_sc_ due to efficient near-surface photon absorption, they also tend to decrease V_oc_, most likely due to enhanced recombination losses at the surface [27]. These findings emphasize the delicate balance required between emitter thickness and dopant concentration. Striking this balance is critical for balancing the numerous performance metrics and achieving an optimized solar cell design.

**Fig. 10:**
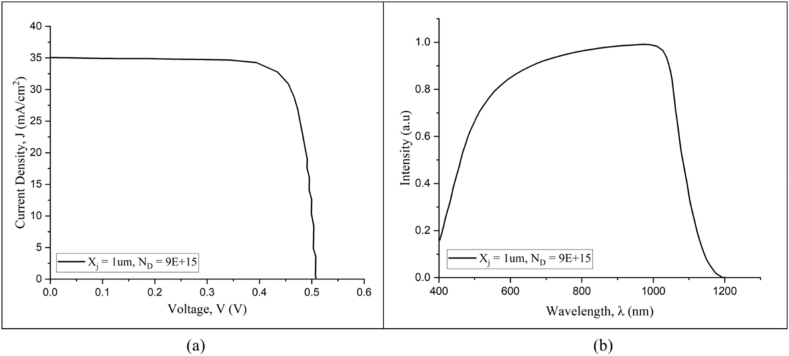
(a) J-V curve and (b) QE spectra of the optimized simulated silicon homojunction solar cell.

The J-V curve and QE spectra, as shown in Fig. 10, provide useful metrics for assessing the performance of the optimized homojunction silicon solar cell, specifically at an emitter thickness of X_j_ = 1 μm and a dopant concentration of N_D_ = 9e+15 cm^−3^. The J-V curve depicts the electrical behavior of a solar cell under illumination, highlighting the interaction between current and voltage. Such curves are critical in determining the efficiency of photovoltaic devices. At the same time, the QE spectra reveal the cell's collection efficiency after converting photon energy into energized electrons at different wavelengths. It reveals how effectively the solar cell utilizes solar radiation across the spectra. Table 4 quantifies the optimized silicon solar cell's performance attributes further. It is critical to note that achieving peak performance does not necessitate each parameter operating at its maximum.

To put it another way, while a high J_sc_ may indicate efficient photon-to-electron conversion, it may also indicate a lower V_oc_ due to increased recombination losses. As a result, achieving a harmonious balance among these parameters is critical for realizing an optimized or the best solar cell design.

## 4 Conclusion

An effort to optimize critical parameters in phosphorus-doped emitters by spin-on doping for silicon homojunction solar cells has been meticulously addressed in this study through a fusion of empirical data and computational simulations. It is found that a quartet of 0.75 ml phosphorus dopant layers applied prior to the drive-in diffusion process strikes the ideal balance. The highest efficiency observed for the in-house fabricated rudimentary sample is 11.75 %, with a J_sc_ of 29.79 mA/cm^2^, V_oc_ of 0.51 V, and FF of 76.92 %. Most importantly, this efficiency is achieved on a pristine surface cell with no texturization, emphasizing the rudimentary nature in terms of the inherent quality and potential of the material and design only. The investigation reveals an intriguing trend in which the initial addition of dopant layers improves efficiency, but an overzealous approach with excessive layers introduces resistive challenges. This discovery warns against the dangers of doping and emphasizes the importance of strategic design considerations. The combinatorial analysis illuminating the interaction between N_D_ and X_j_ for the silicon homojunction solar cell attests to the intricate balance required in semiconductor design. When the emitter thickness, X_j_, is fine-tuned to 1 μm, the efficiency peaks at 14.18 %. This optimal depth emphasizes the intricate harmony between J_sc_, V_oc_, and FF, as well as how minor differences can have a cascading effect on overall cell performance. In the broader context, these findings have implications for the solar cell industry, potentially guiding the design and production of more efficient and cost-effective solar cells.

## Data availability

Data will be made available on request.

## CRediT authorship contribution statement

**Ili Salwani Mohamad:** Writing – review & editing, Writing – original draft, Visualization, Methodology, Investigation, Formal analysis, Data curation, Conceptualization. **Pin Jern Ker:** Writing – review & editing, Validation, Supervision, Investigation. **Puvaneswaran Chelvanathan:** Writing – review & editing, Supervision, Investigation, Formal analysis, Data curation, Conceptualization. **Mohd Natashah Norizan:** Writing – review & editing, Visualization, Validation, Supervision, Project administration, Data curation, Conceptualization. **Yap Boon Kar:** Writing – review & editing, Project administration, Funding acquisition. **Tiong Sieh Kiong:** Writing – review & editing, Project administration, Funding acquisition. **Nowshad Amin:** Writing – review & editing, Validation, Supervision, Resources, Project administration, Investigation, Data curation, Conceptualization.

## Declaration of competing interest

The authors declare the following financial interests/personal relationships which may be considered as potential competing interests: Yap Boon Kar reports financial support was provided by Malaysia 10.13039/501100003093Ministry of Higher Education. If there are other authors, they declare that they have no known competing financial interests or personal relationships that could have appeared to influence the work reported in this paper.
