# Supplementary material for: An experimental investigation of spin-on doping optimization for enhanced electrical characteristics in silicon homojunction solar cells: Proof of concept
Source: Heliyon. 2024 May 14;10(11):e31193. doi: 10.1016/j.heliyon.2024.e31193 (PMC11140604; doi:10.1016/j.heliyon.2024.e31193)
Supplement: Multimedia component 1 [file mmc1.docx]

Supplementary Data


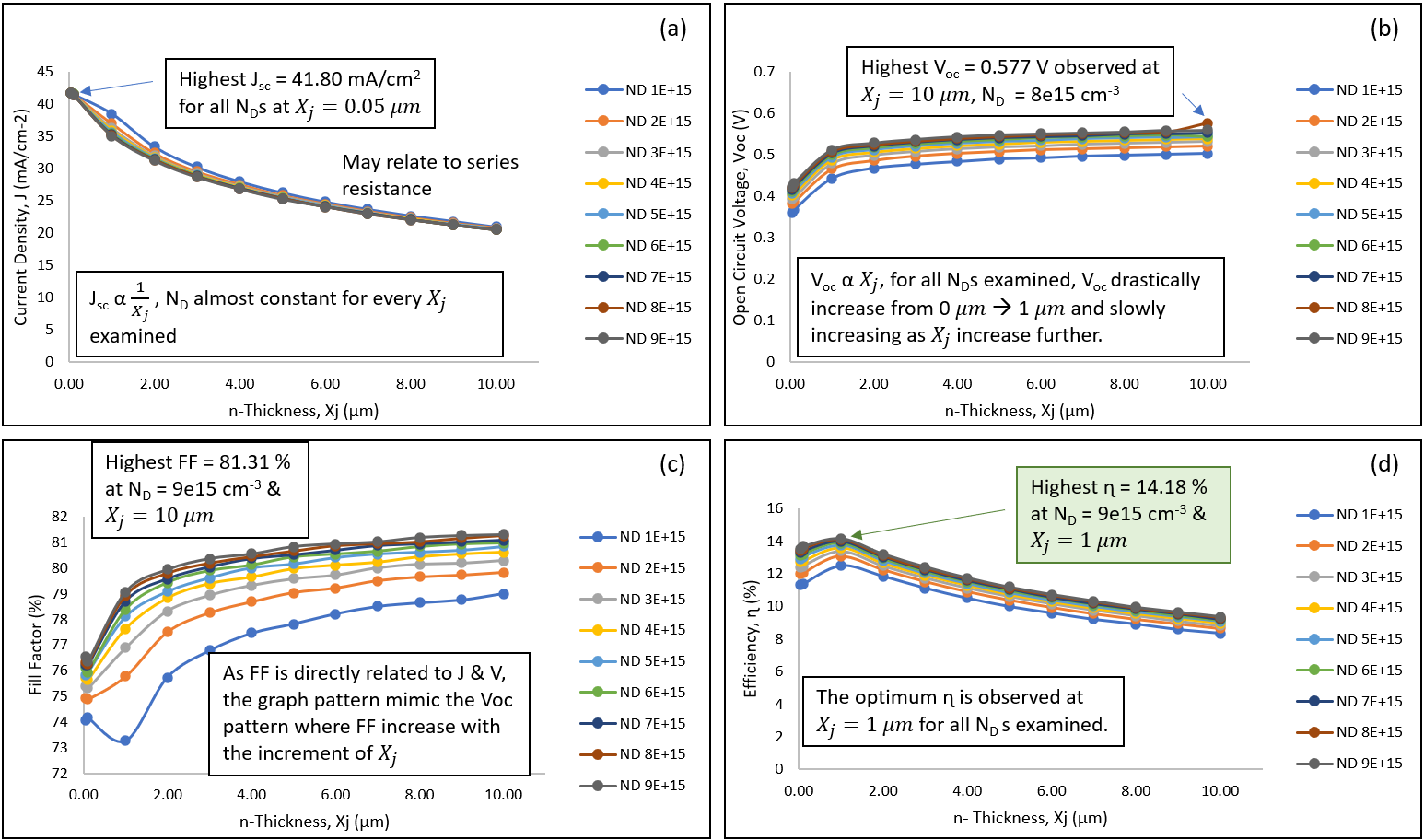


Figure 1: Electrical parameters output on the combinatorial analysis for simulated homojunction silicon solar cell (a) J_sc_ for X_j_ & N_D_ variations (b) V_oc_ for X_j_ & N_D_ variations (c) FF for X_j_ & N_D_ variations (d) Efficiency for X_j_ & N_D_ variations

Figure 1 depicts the electrical parameter results from the simulation of a homojunction silicon solar cell, with variations in emitter thickness and carrier concentration. The combinatorial analysis variations include emitter thickness (X_j_) of 0.05 μm, 0.1 μm, 1 μm to 10 μm (1 μm step size) and emitter carrier concentration (N_D_) ranging from 1E+15 cm^-3^ to 9E+15 cm^-3^ (1E+15 cm^-3^ step size). A decreasing trend in current density (J_sc_) is observed for all carrier concentrations tested as the thickness increases with the highest J_sc_ = 41.80 mA/cm^2^ at X_j_ = 0.05 μm. In contrast, V_oc_ abruptly increases when X_j_ increases from 0 to 1 μm and marginally increases as the thicknesses increase. Highest V_oc_ recorded in this work is 0.577 V at X_j_ = 10 μm and N_D_ = 8E+15 cm^-3^. Fill factor (FF) represents show ‘‘difficult’’ or how ‘‘easy’’ the photogenerated carriers can be extracted out of a photovoltaic device. The ideal value for FF is unity (100%). Figure 1(c) reveals that for all thickness tested, FF is best at high N_D_. FF is illustrated to be increasing with the increment of junction depth and the best FF= 81.31 % is recorded at N_D_ = 9E+15 cm^-3^ and X_j_ = 10 μm. Figure 1 (d) summarises the findings and proved that the highest efficiency does not need all electrical parameters to be at its maximum as its relation of ɳ = (J_sc_ x V_oc_ x FF) / P_in_. The highest efficiency recorded for this combinatorial analysis is observed to be 14.18 % at X_j_ = 1 μm and N_D_ = 9E+15 cm^-3^.

Junction depth can affect the series resistance in the following ways;

Shallow Junction: With a shallow junction depth, the distance that carriers need to travel to reach the contacts is reduced. This shorter path reduces the series resistance, as there are fewer obstacles for carriers to encounter during transport.

Deep Junction: Conversely, a deeper junction depth increases the distance carriers need to travel before reaching the contacts. This longer path results in higher series resistance due to increased resistive losses in the material and contact regions.
